# Computational Biology in Costa Rica: The Role of a Small Country in the Global Context of Bioinformatics

**DOI:** 10.1371/journal.pcbi.1000040

**Published:** 2008-03-14

**Authors:** Edgardo Moreno, Bruno Lomonte, José-María Gutiérrez

**Affiliations:** 1 Programa de Investigación en Enfermedades Tropicales, Escuela de Medicina Veterinaria, Universidad Nacional, Heredia, Costa Rica; 2 Instituto Clodomiro Picado, Facultad de Microbiología, Universidad de Costa Rica, San José, Costa Rica; University of California San Diego, United States of America

## Introduction

The successful development of high throughput methods for DNA sequencing, transcriptomics, proteomics, and other –omics, has contributed to the emergence of novel possibilities for the examination of complex biological systems through computational analysis. These fields have witnessed unprecedented advances in high income countries. Nevertheless, the role of other nations needs to be examined in order to delineate their contribution within the global context of bioinformatics. Previous articles have focused on the expansion of Computational Biology in Brazil and Mexico [1],[2], two of the largest Latin American countries, and which have shown political commitment to foster their scientific development. Costa Rica is a small Central American country with a population of 4 million, with its territory 164 and 38 times smaller than Brazil and Mexico, respectively. Thus, it is interesting to visualize the possibilities and challenges of this low-income country in the context of the global bioinformatics endeavor. (For author information, see Box 1.)

**Box 1. Author Biographies**

**Edgardo Moreno (EM), Bruno Lomone (BL), and José-María Gutiérrez (JMG)** received their B.Sc. degrees in Microbiology and Clinical Chemistry at the University of Costa Rica. They obtained their Ph.D.s at the University of Wisconsin-Madison (EM), the University of Göteborg, Sweden (BL), and Oklahoma State University (JMG). Besides their formal training, the three of them have obtained fellowships to perform research in laboratories in Brazil, México, France, Germany, and the United States, maintaining strong collaborations with valuable foreign research groups. Upon their return to Costa Rica, they were appointed to the staffs of the School of Veterinary Medicine, National University (EM), and the Clodomiro Picado Institute, School of Microbiology, University of Costa Rica (BL and JMG), where they have been teaching Immunology, Cell Biology, Cell Pathology, and Biochemistry, at both graduate and undergraduate levels.

EM's main research interests have been in the understanding the cross-talk between *Brucella* intracellular pathogens and their animal host cell and the host adaptation and evolution of this endoparasitic bacterium. BL has investigated the structural and functional characteristics of snake venom toxins, contributing to the identification of the molecular regions responsible for the disruption of muscle cell plasma membrane. His interests also include the immunochemical characterization of snake venom components, modeling, and the proteomic analysis of venoms from Central American snakes. JMG's interests have focused mostly on the experimental pathology of snakebite envenomation and regeneration of tissues after snake venom-induced damage. He has also been devoted to the improvement of the technology for antivenom production at the local and global levels, participating in a WHO-lead initiative to improve antivenom production worldwide.

EM, BL, and JMG have actively collaborated together over many years, and have participated in a number of regional projects promoting long-lasting academic and scientific alliances between research groups and institutions from Central America and Sweden, in what is currently known as the *NeTropica* network. They have also contributed to the consolidation of local and regional graduate programs based in Costa Rican public universities. In addition to their engagement in university activities, the three of them are members of the National Academy of Sciences of Costa Rica.

## 

At first glance, it is evident that the role of Costa Rican scientists is not to compete and struggle for the growth of these high throughput technologies, but instead to use them to investigate specific biological models and to provide additional data that could help decipher the information encrypted in the contemporary cybernetic “Rosetta Stones”. There is a growing consensus that significant biological questions are seldom answered by moving from the kilobases to the megabases of sequences, or by describing all the proteins or transcripts in a particular system. The enormous volume of data generated by genomics, proteomics, and transcriptomics demands meticulously described phenotypes, and a detailed understanding of the biological and ecological scenarios, to contextualize all the “linear” information provided by high throughput methodologies. Within this perspective, it is worth noting that in spite of its small size, Costa Rica harbors close to 5% of the world's biodiversity, not taking into consideration its oceanic littorals, which are extremely rich in genotypes, as revealed by the survey performed near to the Cocos Island by The Sorcerer II, in which Costa Rican investigators participated [3]. More than one quarter of the country's territory comprises well-known protected areas, mainly national parks and reserves. Such vast biodiversity includes a rich substrate for a community of both local and foreign scientists devoted to Systematics, Ecology, and Evolutionary Biology, who feed databases with highly valuable information [4]. Other scientists have taken advantage of the fact that Costa Rica was founded by a small number of families (in 1900, the population was about 180,000), which generated genetically isolated groups that are amenable for studies linking human DNA sequences with specific phenotypes [5]. Additionally, there are good and reliable databases on the biogeography of Costa Rica, greatly contributing to sustaining ecological and epidemiological investigations that provide information that can be associated with specific and well-defined systems, such as pathogen phylogenies [6] or metagenomic studies [7]. From the global perspective, then, Costa Rica constitutes an interesting case of a biologically rich small country that can be analyzed for the possibilities of contributing to the growing field of Computational Biology. This essay describes the examples of three Costa Rican institutions that participate in international consortia and collaborate with research centers in the analysis of data generated by high throughput methods, and illustrates some of the major problems confronted.

## From Linnaeus to DNA Barcodes

What do *Aedes aegypti* and *Annona muricata* have in common? The most likely immediate response by expert medical entomologists would be “nothing”, since the first species is a culicid mosquito capable of transmitting dengue and yellow fever, while the second is a delicious tropical fruit, highly appreciated by many animals, including humans. However, a broader analysis and a more acute examination by devoted naturalists and taxonomists would tell us that both species inhabit the same geographical regions in Central America and that they were classified and named by the great naturalist Carolus Linnaeus (1707–1778) almost 250 years ago. Biologists and botanists searching for bioactive compounds in plants would answer that the mosquito larvae do not develop in stagnant water in the flowers, fruits, or holes of this annona tree, because it contains large quantities of flavonoids which exert potent inhibitory and lethal activity against many insects, including larvae of the dangerous *A. aegypt*i. Notably, and coming back to the subject of this essay, lay men and scientists realize that all these pieces of information and relationships can be extracted from databases assembled by the National Biodiversity Institute of Costa Rica (INBio) by performing a few “mouse clicks” (http://www.inbio.ac.cr).

INBio was established in 1989 to obtain information on the country's biological diversity and to promote its sustainable use. Although INBio is a Costa Rican initiative in terms of its scope and staff, it is part of an international effort aimed at integrating knowledge of inventory and monitoring species and ecosystems, conservation of species and their habitats, education on the country's biodiversity within the global context, bio-prospecting, and biodiversity informatics. INBio has developed an information system named Atta that supports the core information obtained and processed by the institution in collaboration with many scientists (http://atta.inbio.ac.cr/attaing/atta03.html). Atta facilitates the processes of gathering, managing, generating, and disseminating information on Costa Rican biodiversity. The system maintains a relational database with over two million records, each corresponding to a single specimen linked to a unique barcode physically attached to it. This information includes a complete description of the specimen, its biological context, and modules for generating, consulting, editing, and analyzing taxonomic, geographic, and ecological information, and potential uses of the species. One of Atta's modules, named UBIs, allows collaborators to submit species descriptions by means of an electronic publication process. INBio is part of the “Barcode for Life Initiative” (BOLI), a consortium devoted to the generation of short sequences of selected genes from a standardized position in the genome which are then used as DNA barcoding fingerprints for identifying species, analogous to black bar strips used in stores to identify goods. The information is then linked to databases such as Atta, which is used to contextualize the species identification. This system has proven effective in assigning organisms to known species using only a tiny piece of tissue, often belonging to valuable specimens kept in museums, to discover new variations (biotypes) within a species, and to document the biodiversity of poorly known taxonomic groups or inadequately sampled geographical areas. INBio was one of the first institutions devoted to the inventory of biodiversity in the world, and is promoting the use of Computational Biology for relating data generated by high throughput methods with phenotypes, ecosystems, and evolutionary and functional studies.

## Integrating Genomics and Proteomics with Pathogen Life Styles

The Tropical Disease Research Program (PIET) of the National University of Costa Rica is devoted to the study of relevant microbial pathogens. This program approached the subject of Computational Biology two decades ago, by analyzing RNA sequences of pathogenic bacteria and viruses, generating phylogenies, and relating them to specific biological and ecological systems [6],[8],[9]. One significant study determined that ecological rather than temporal factors dominated the evolution of vesicular stomatitis virus, an important veterinary pathogen. This is the first report describing the dispersion of different viral strains within specific ecological systems and represents a computational stochastic model for the dispersion of viral genotypes [6]. Phylogenomics, which constitutes the integration of genomic and evolutionary analyses, became a powerful analytical tool for presenting key hypotheses on the genome reduction of animal alpha-proteobacteria living in close association with eukaryotic cells, as well as on the origin of chromosomes from plasmids [8],[9], a proposal that was later supported by analysis of complete genomic sequences of these intracellular bacterial pathogens [10],[11]. More recently, researchers of PIET, in collaboration with investigators from Caprion Proteomics, a Montreal-based pharmaceutical company, have participated in comparative proteomic studies of the intracellular bacterial parasite *Brucella* and its host cells. One study on the quantitative proteomics of outer membrane fragments of wild type *Brucella* and the corresponding non-virulent mutant strains in a two-component sensor–regulator system revealed extensive modulation of a large number of proteins by this system, affecting mainly the cell surface, substrate uptake, and stress responses [12]. These multiple effects, influenced by the two-component sensor–regulator system, evidenced that *Brucella* virulence depends on complex regulatory networks and not on the addition of hitherto undefined discrete virulence factors. This study constitutes a novel quantitative proteomic approach of isolated bacterial fractions, allowing the deciphering of relationships between the location of proteins, their function, and their regulation. Interestingly, this model has implications in other related parasitic alpha-proteobacteria harboring similar orthologous systems essential for parasitism or endosymbiosis. Indeed, investigators from The Virginia Bioinformatics Institute, a premier center in Computational Biology, are presently collaborating with PIET researchers to study the outer membrane fragments of wild type *Sinorhizobium* and the corresponding mutants, a plant endosymbiont closely related to *Brucella*. These examples illustrate how pathogen models developed in research centers from small countries are valuable for further genomic, proteomic, or transcriptomic analyses. Moreover, joining efforts with institutions harboring high throughput technology constitutes an adequate strategy to annotate and give meaning to linear data on pathogenic organisms, which has to be reliable for further computational analysis.

## Snake “Venomics”: An Exciting Source of Biological Novelties

**Figure 1:**
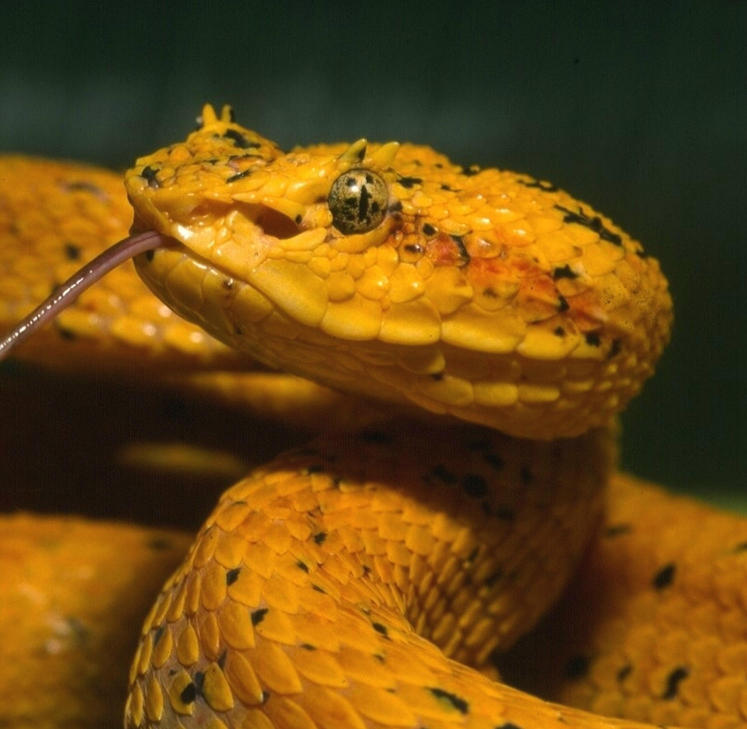
Adult *Bothriechis schlegeli*, an Arboreal Snake Abundant in Costa Rican Rain Forests, Whose Venom Composition Has Been Analyzed by Proteomics.

Costa Rica harbors a rich herpetofauna, with more than 65 genera and 137 species of snakes, of which 22 are highly poisonous (Figure 1). The Clodomiro Picado Institute (ICP), of the University of Costa Rica, is a research center devoted to the study of snake venoms and to the production of therapeutic antivenoms utilized in the region. Poisonous snakes secrete venoms that contain hundreds of bioactive toxic proteins, coded in genes that have evolved by a process known as “accelerated evolution” during 60–80 million years [13],[14]. Bioinformatics combined with high throughput technologies have just begun to unravel the fascinating pathways by which ancient genetic scaffolds evolved to code for potent poison snake toxins that drastically affect a variety of key physiological processes of prey animals [15]. These diverse bioactivities are relevant from the basic as well as from the medical point of view, since new molecular tools and clinically useful drugs may be developed from studies on venom proteins. A number of toxic proteins, among them metalloproteinases, phospholipases A_2_, serine proteinases, and lectin-like components, have been purified from Costa Rican snake venoms, and their toxic mechanisms have been deciphered. Investigators from the ICP have initiated an ambitious collaboration with researchers at the Biomedical Institute of Valencia, Spain, on “venomics” [16], aimed at resolving the proteomes of Costa Rican snake venoms. The conspicuous radiation that occurred in viperid snakes in Central America, resulting in many species classified in eight different genera within a limited geographic area, constitutes an excellent evolutionary arena for the diversification of venom components and the concomitant appearance of novel toxic and pharmacological activities in these secretions. This information is, therefore, a valuable source of data [17],[18] which will bring insights into the evolutionary, phylogenetic, and functional relationships of these amazing toxic proteins. Additionally, snake venomics is being complemented with proteomic analysis of venom proteins recognized by antibodies from hyperimmune sera, thus paving the way for a more detailed characterization of their neutralizing scope and for the design of more effective antivenoms. Computational tools, in combination with the analysis of the toxicological and pharmacological activities of venom components, will provide a unique framework for understanding the pathology of envenomations and for identifying new lead compounds for pharmaceutical investigations.

## Breaking Language Barriers

Although these three examples illustrate a successful integration of Biology with Computational Sciences, there is still a long way to go before a fluent cross-talk between these two disciplines consolidates in specialized research centers in Costa Rica. As in other Latin American countries, most Costa Rican investigators dedicated to computational interfaces are not familiar with the issues of life sciences; and biologists, on the other hand, have limited knowledge in programming and computer modeling. Exceptionally, few taxonomists, evolutionists, molecular biologists, biochemists, and microbiologists, confined to the molecular codes of life systems and the binary language of cybernetics, reinvented themselves as computer scientists devoted to solving biological questions. Concomitantly, only few local computer scientists have been interested in biological topics. Moreover, none of the undergraduate or graduate careers related to computational or biological sciences offered by the Costa Rican public educational institutions contemplate an integrated curricula. This is relevant since close to 95% of the country's research is generated by academicians working in public universities. As a consequence, students interested in Computational Biology have to struggle against rigid programs that slow down the emergence of this “new discipline” in the country.

A strategy for promoting the development of Computational Biology in Costa Rica demands the integration of dispersed efforts by local scientists who have already gained experience in the analysis of complex data by means of bioinformatics. One of the initial actions should be the introduction of topics related to high throughput technologies, biological data mining, epidemiological predictions, molecular modeling, and other related subjects to the curricula of university programs dealing with biological or computational sciences. The integration of research actions by the public universities (http://www.conare.ac.cr/), the physical proximity of scientific centers, mostly located in the central valley of Costa Rica, and the frequent interactions of academic groups may facilitate these efforts. The recent development of a computer “cluster” at the University of Costa Rica is a step forward, but further local and international support is urgently required.

In addition to upgrading the universities' curricula, the country's foremost strategy has to be to concentrate on educating young people in renowned international institutions with programs devoted to Computational Biology. Upon students' return to Costa Rica, clear political actions are required to strategically appoint these “newcomers” to local research centers, thus promoting the consolidation of this field, the training of new students, and the process of fundraising. Although a “brain drain” would be inevitable to some extent, Costa Rican research units must maintain efforts to educate people abroad while strengthening the conditions for their productive return and reinsertion into their home country [19].

## Bringing Funds to Limbo

Research funding in Costa Rica is scarce, especially for basic sciences, and, as in other low-income countries, professional researchers cannot depend exclusively on national resources. For many international agencies devoted to supporting low-income nations, Costa Rica is not considered for funding since, according to health indexes and other social parameters, the country is envisioned as “advanced”. On the other hand, our scientific devolvement lacks the critical mass, strength, and consistency to compete for robust research grants abroad. Certainly this “limbo situation” is a handicap for most scientists working in Costa Rica, and the “small is beautiful” slogan of Ernst F. Schumacher (1911–1977), which has been a good strategy for promoting tourism to this neotropical region, has not been efficient for capturing international research funds.

Although computational sciences have to struggle with this state of affairs, and compete for the limited resources available, in recent years the Ministry of Science and Technology, the Council for Scientific Research (CONICIT), a few private companies, and the public universities of Costa Rica have considered computational disciplines a priority within the local scientific agenda. Surprisingly, however, very few projects involving Computational Biology have been presented and even fewer have been approved (http://www.conicit.go.cr/conicit/). Evidently, this underscores a lack of understanding of the relevance of this discipline in Costa Rica. It does not mean, however, that efforts in this area are absent, as previously illustrated, but merely indicates that Computational Biology has been masked by the long-established scientific subjects, and that more attention is required to promote this field, through innovative strategies involving local and international actors.

## Concluding Remarks

After one quarter of a millennium of the taxonomic “baptism”, using binomial nomenclature, of the vicious *A. aegypti* (meaning “Egyptian grave”) by Linnaeus, scientists have sequenced the genome of this mosquito, the human genome, and those of dengue virus strains. However, dengue still remains a dangerous malady whose impact is steadily increasing in many regions. With the prospect of global warming, the distribution range of this vector is likely to increase in the near future, with the consequent impact in the incidence of dengue in areas where it has been absent. The question of how to link the genomic information in order to generate coherent models that could contribute to the control of the mosquito and the infection, constitutes a challenge that demands high-quality biological, phylogenetic, epidemiological, and ecological investigations. Certainly, Costa Rica requires more research on this topic [20], as well as in many other areas that could be helped in generating adequate models by using Computational Biology. The complexity of the biological and epidemiological phenomena associated with conservation and disease control urges concerted and integrated efforts along different lines. Within the context of Costa Rica, this task requires a significant strengthening of the local scientific community, the consolidation of groups that have developed a critical mass of scientists and expertise, and the fostering of dynamic interactions between local groups and the international scientific community. The examples of successful efforts in the study of the biological diversity in Costa Rica should be appreciated and extended to other subjects, through active collaborative projects. Definitely, there is a place for small scientific communities in the global efforts to use Computational Biology for understanding, appreciating, and using the biodiversity of the planet, as well as for strengthening our potential to investigate genetic diseases and the natural history and life cycles of vectors and pathogens. These comprehensive efforts should be based on a philosophy of sustainability, equity, and solidarity, which necessarily includes a broad free access to scientific information.
